# Two new species of the *Exocelina ekari* group from New Guinea with strongly modified male antennae (Coleoptera, Dytiscidae, Copelatinae)

**DOI:** 10.3897/zookeys.960.55007

**Published:** 2020-08-17

**Authors:** Helena Shaverdo, Suriani Surbakti, Bob Sumoked, Michael Balke

**Affiliations:** 1 Naturhistorisches Museum Wien, Burgring 7, 1010 Vienna, Austria Naturhistorisches Museum Vienna Austria; 2 Department of Biology, Universitas Cendrawasih, Waena, Papua, Indonesia Universitas Cendrawasih Waena Indonesia; 3 Walian 2, Tomohon Selatan, N Sulawesi 95439, Indonesia Unaffiliated Tomohon Indonesia; 4 SNSB-Zoologische Staatssammlung München, Münchhausenstraße 21, D-81247 Munich, Germany and GeoBioCenter, Ludwig-Maximilians-University, Munich, Germany SNSB-Zoologische Staatssammlung München Munich Germany

**Keywords:** Australasia, distribution, *
Exocelina
*, systematics, new species

## Abstract

Two new species of the genus *Exocelina* Broun, 1886: *E. athesphatos***sp. nov.** and *E. tsinga***sp. nov.** are described from New Guinea and placed into the *E. ekari* group based on the structure of their male genitalia. The two species are very similar with respect to their external morphology and characterised by almost identical, strongly modified male antennae. However, they can easily be separated by the shape and setation of the median lobe and paramere. Based on morphological similarity and results of a molecular phylogenetic analysis, we suggest these are sister species. Both of them have been collected on the southern slopes of the Central Range (the spine of New Guinea), with a distance of ca. 380 km straight line between the collecting localities.

## Introduction

Two new species of the genus *Exocelina* Broun, 1886 discovered on the southern slopes of the New Guinea Central Range are introduced. Having a discontinuous outline of the median lobe of the male genitalia, both belong to the largest *Exocelina* species group, *E. ekari* group. To date, this group contains 54 species (including the two new species) endemic to New Guinea (Balke 1998; Shaverdo and Balke 2019; Shaverdo et al. 2005, 2012, 2014, 2016). Including the results of this paper, 142 species of *Exocelina* are now described from New Guinea and 199 species worldwide (Shaverdo and Balke 2019; Shaverdo et al. 2019; Balke and Ribera 2020; Nilsson and Hájek 2020). As in most of our previous papers on the genus, all species data will be presented on the species-id.net portal automatically created by ZooKeys with the publication of this paper.

## Materials and methods

The present work is based on material from the following collections:

**KSP** Koleksi Serangga Papua, at the Biology Department of Universitas Cenderawasih (UNCEN), Waena, Papua, Indonesia;

**MZB**Museum Zoologicum Bogoriense, Cibinong, Indonesia.

Our methods follow those described in detail in our previous articles (Shaverdo et al. 2012, 2014; Shaverdo and Balke 2014). The terminology to denote the orientation of the genitalia follows Miller and Nilsson (2003). All specimen data are quoted as they appear on the labels attached to the specimens. Label text is cited using quotation marks; comments in square brackets are ours. The following abbreviations were used: TL (total body length), TL-H (total body length without head), MW (maximum body width).

## Species descriptions

### Exocelina athesphatos sp. nov.

Taxon classificationAnimaliaColeopteraDytiscidae

5C7CE25B-1310-50B0-8E24-90D22ACAABDE

http://zoobank.org/B42F6969-FA5C-48A9-A542-ECDCC0A25CB4

**Figure 1.**
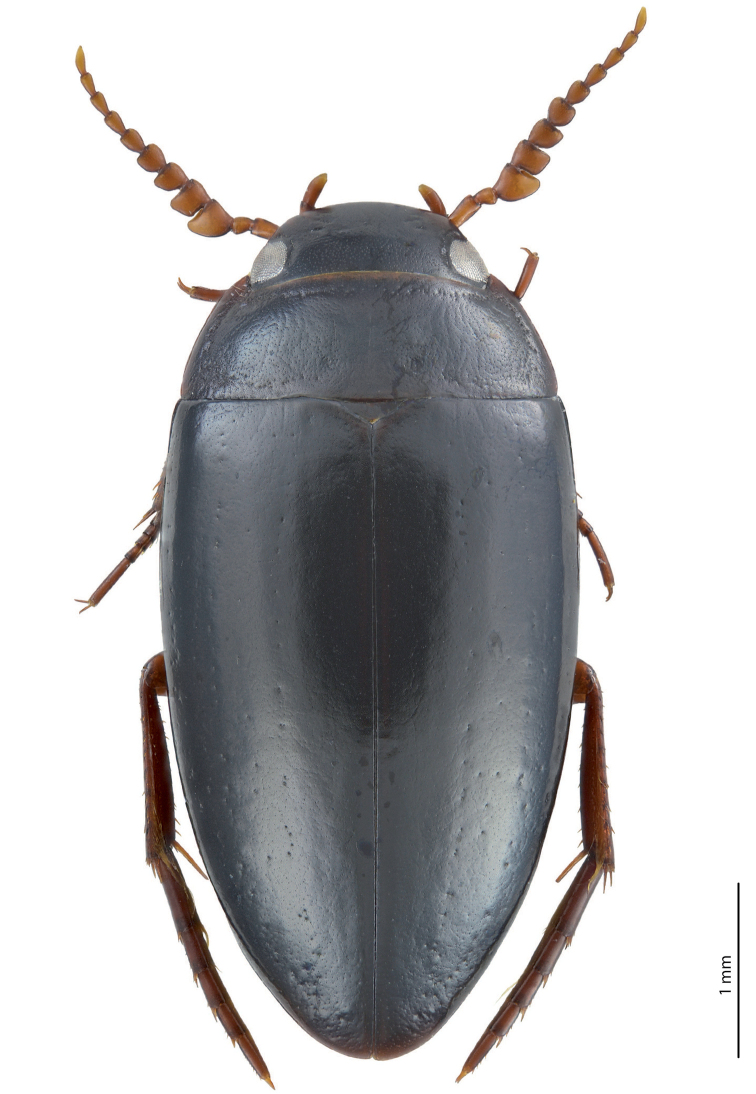
Habitus and colouration of *Exocelina athesphatos* sp. nov., holotype.

**Figure 2.**
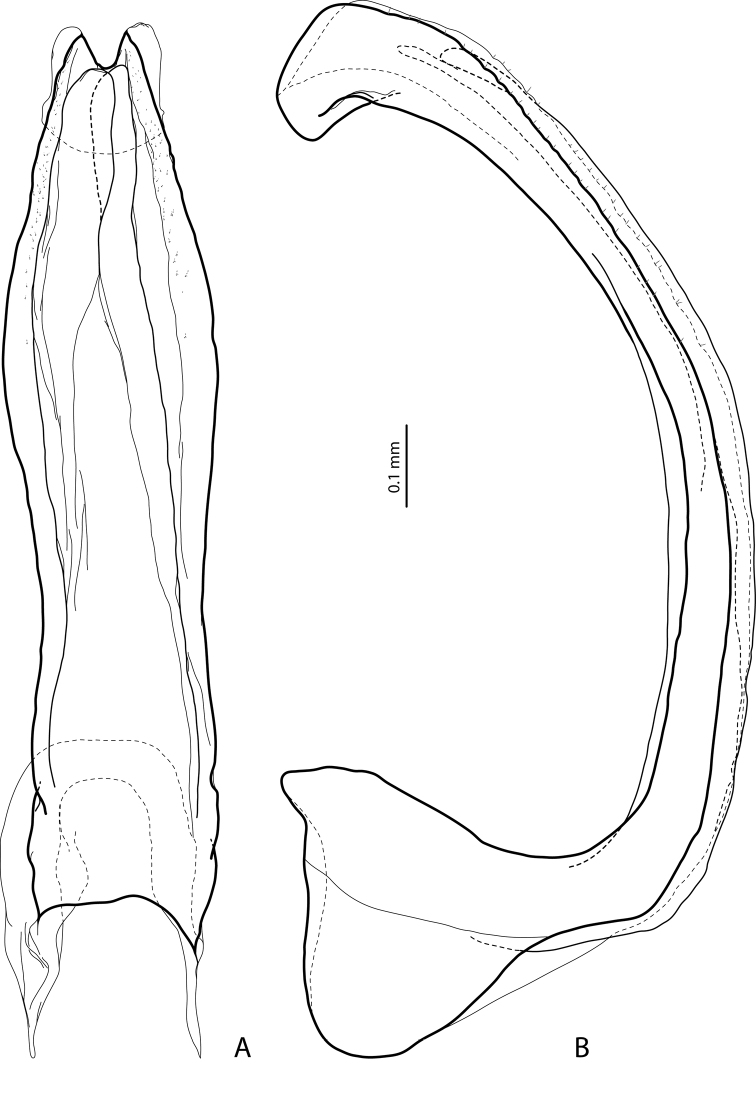
*Exocelina athesphatos* sp. nov., paratype **A** median lobe in ventral view **B** median lobe in lateral view.

**Figure 3.**
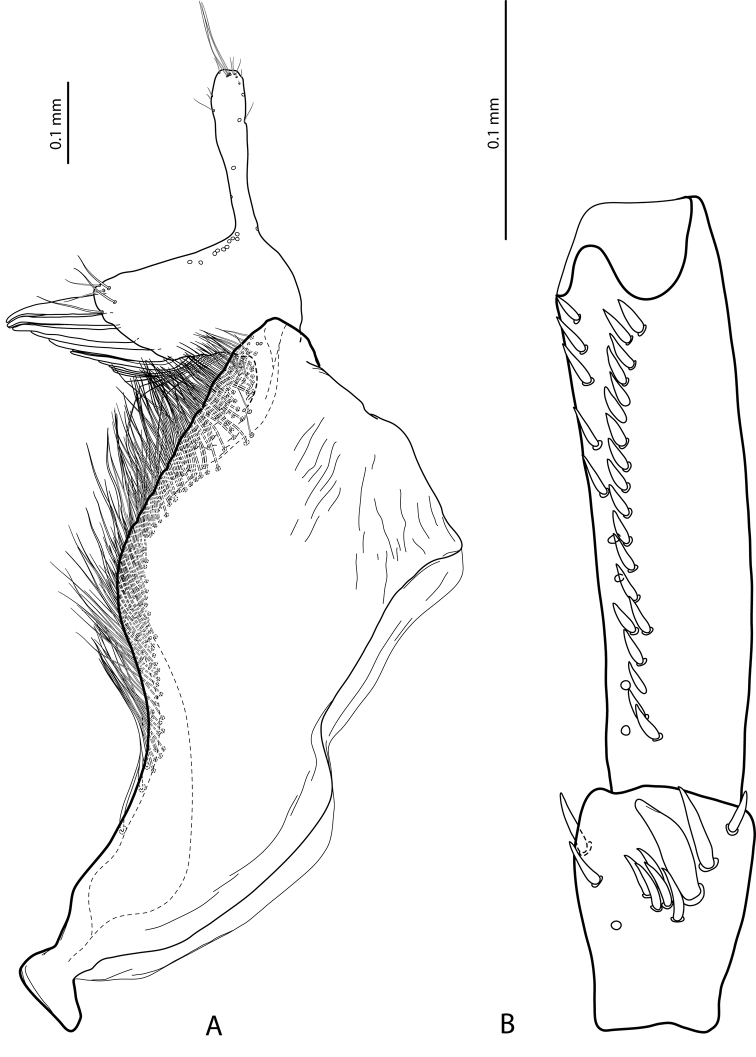
*Exocelina athesphatos* sp. nov., paratype **A** right paramere in external view **B** right male protarsomeres 4–5 in ventral view.

**Figure 4.**
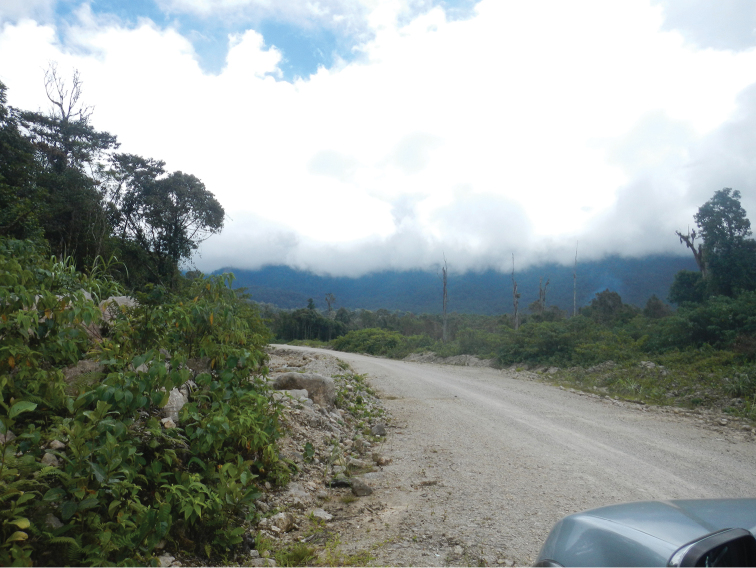
Dirt road from Ok Sibil to Ok Bap.

Figures 1 , 2 , 3 , 4 , 8A , 9A

#### Type locality.

Indonesia: Papua Province: Pegunungan Bintang Regency, near Ok Bap, 04°49'28.6"S, 140°24'47.0"E, 1,961 m a.s.l.

#### Type material.

***Holotype***: male “Indonesia: Papua, nr Ok Bab [sic!], 1961 m, 8.vi.2015, -4.82460033148527, 140.413050251081, Sumoked” (MZB). ***Paratypes***: 16 males, 9 females with the same label as the holotype (MZB, KSP).

#### Description.

***Body size and form***: Beetle medium-sized: TL-H 4.3–4.8 mm, TL 4.85–5.4 mm, MW 2.3–2.5 mm (holotype: TL-H 4.6 mm, TL 5 mm, MW 2.4 mm), with oblong-oval habitus.

***Colouration***: Dorsally piceous, sometimes with dark brown posterior part of head, middle and lateral parts of pronotum, and usually with dark brown elytral sutural lines; head appendages yellowish red, legs yellowish red to reddish brown (Fig. 1). Teneral specimens paler, reddish brown.

***Surface sculpture***: Shiny dorsally, with inconspicuous to fine, distinct punctation and weakly impressed microreticulation. Head with dense punctation (spaces between punctures 1–2 times size of punctures), evidently finer and sparser anteriorly; diameter of punctures almost equal to diameter of cells of microreticulation. Pronotum with sparser and finer punctation than head. Elytra with very sparse and fine punctation, sometimes inconspicuous. Microreticulation of elytra weakly impressed, in some specimens slightly stronger. Pronotum and especially head with stronger microreticulation. Metaventrite and metacoxae distinctly microreticulate, metacoxal plates with longitudinal strioles and transverse wrinkles. Abdominal ventrites with distinct microreticulation, strioles, and very fine and sparse punctation.

***Structures***: Pronotum with distinct, relatively narrow lateral bead. Base of prosternum and neck of prosternal process with distinct ridge, slightly rounded anteriorly. Blade of prosternal process lanceolate, relatively broad, slightly convex, with distinct lateral bead and few setae. Abdominal ventrite 6 broadly rounded or almost truncate, with elongate medial impression.

***Male***: Antenna strongly modified (Figs 1, 8A): antennomere 2 strongly reduced, antennomeres 3 and 4 strongly enlarged (antennomere 3 the largest), antennomeres 5 and 6 distinctly enlarged, antennomeres 7–10 stout. Pro- and mesotarsomeres 1–3 dilated. Protarsomere 4 slightly dilated, with anterolateral angle shortly expanded (not visible in Fig. 3B due to a wrong angle, but evident for *E. tsinga* sp. nov. in Fig. 7B; the species are similar in this character) and with large, thick, slightly curved anterolateral hook-like seta. Protarsomere 5 ventrally with anterior row of 18 and posterior row of 9 short, thick, pointed setae (Fig. 3B). Median lobe long and slender, with slightly discontinuous outline (see in apical part), enlarged and thickened apex, and with extremely small, fine, sparse setae distally on lateral margins; apex distinctly curved downwards in lateral view and in ventral view, deeply concave, with divergent sides (Fig. 2). Paramere with very deep dorsal notch, separating subdistal part; subdistal part is very large, broad, with fringe of six or seven very broad, flattened setae and more numerous thin, fine setae; proximal setae numerous, dense, thin, much more inconspicuous than subdistal (Fig. 3A). Abdominal ventrite 6 with relatively deep, elongate medial impression forming two small tubercles on both sides subapically and with 14–18 lateral striae on each side (Fig. 9A).

***Female***: Antennae and pro- and mesotarsi not modified. Abdominal ventrite 6 more rounded apically, with shallow elongate medial impression, without tubercles and lateral striae.

#### Affinities.

Based on shape of the modified male antennae and presence of pronotal bead, the new species can be placed close to the *E. polita* species complex in the identification key. However, this is not a monophyletic group. According to a molecular phylogenetic analysis (Toussaint et al., in preparation), the two new species are sister species, on their own separate branch within the *E. ekari* group.

*Exocelina athesphatos* sp. nov. is similar to *E. utowaensis* Shaverdo, Hendrich & Balke, 2012 in modifications of the abdominal ventrite 6 and general shape of the median lobe and paramere. But the new species distinctly differs from it in larger size (TL-H 3.4–3.8 mm in *E. utowaensis*), having pronotal bead (absent in *E. utowaensis*) and strongly modified male antennae (simple in *E. utowaensis*). Additionally, in general shape of the median lobe and paramere as well as in the relatively narrow pronotal bead, the new species resembles *E. oceai* Shaverdo, Hendrich & Balke, 2012, which is, however, much smaller (TL-H 3.35–3.8 mm) and has simple male antennae. For comparison with *E. tsinga* sp. nov. see below.

#### Distribution.

Indonesia: Papua Province. The species is known only from the type locality.

#### Habitat.

The specimens were collected from small puddles, in roadside ditches besides a dirt road (in Fig. 4 at the left hand side). The beetles have been presumably been washed into these ditches from small forest creeks during heavy rainfalls.

#### Etymology.

The species name *αθέσφατος* derives from Greek, meaning “unadulterated, pure”. The name is an adjective in the nominative singular.

### Exocelina tsinga sp. nov.

Taxon classificationAnimaliaColeopteraDytiscidae

8B770EF3-2480-545B-B610-ED488E6E7DEC

http://zoobank.org/F1184304-DE9D-45AA-936E-17300B34EF25

**Figure 5.**
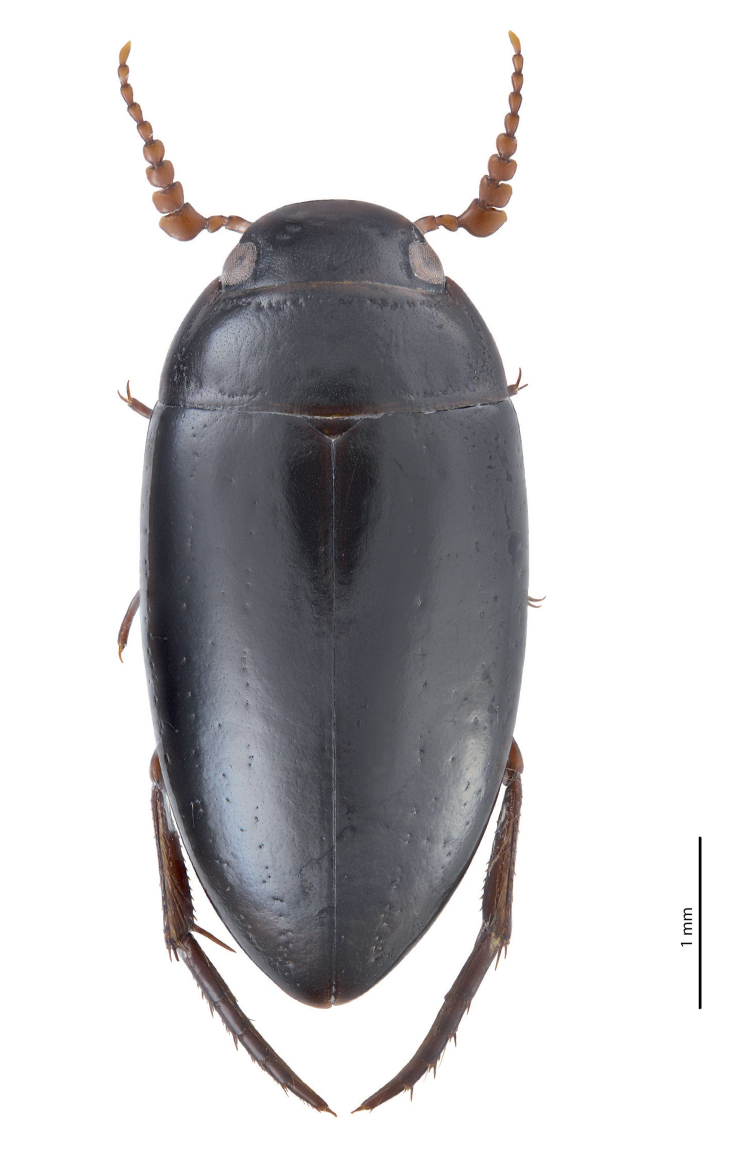
Habitus and colouration of *Exocelina tsinga* sp. nov., holotype.

**Figure 6.**
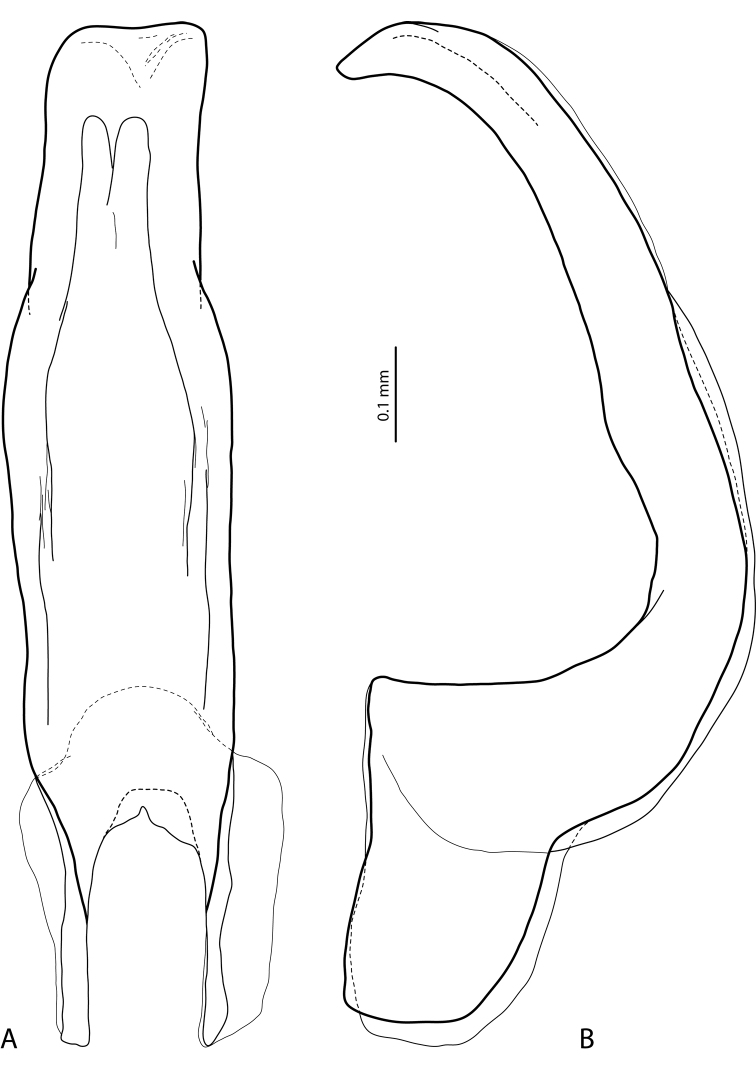
*Exocelina tsinga* sp. nov., paratype **A** median lobe in ventral view **B** median lobe in lateral view.

**Figure 7.**
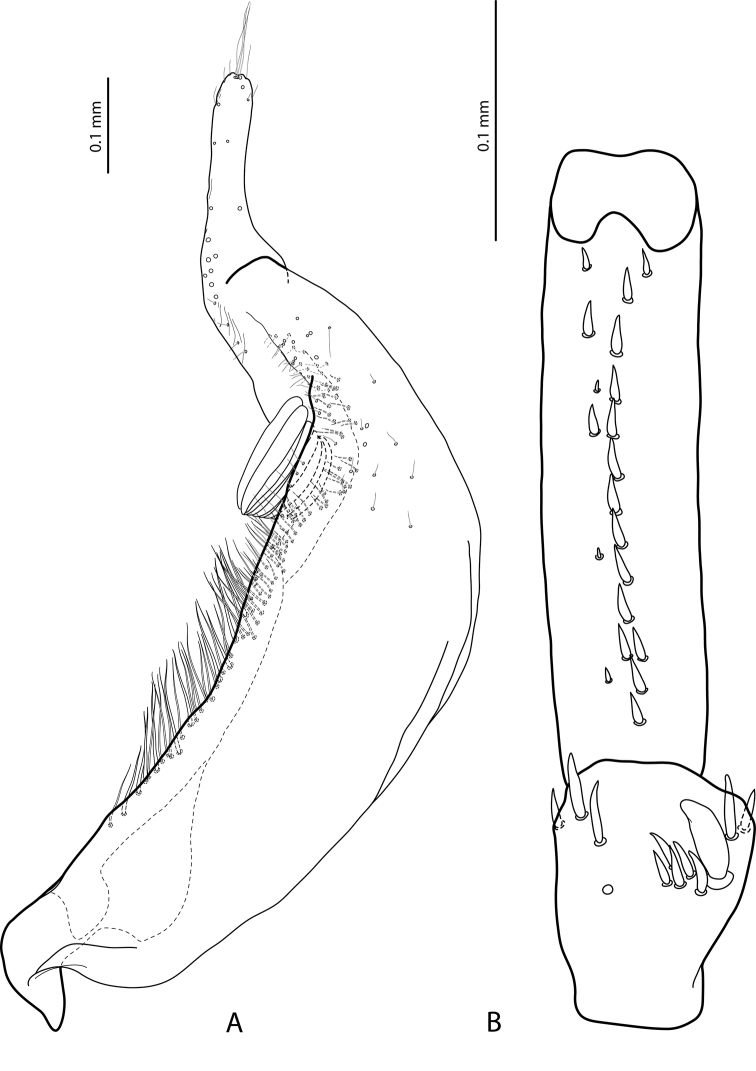
*Exocelina tsinga* sp. nov., paratype **A** right paramere in external view **B** right male protarsomeres 4–5 in ventral view.

**Figure 8.**
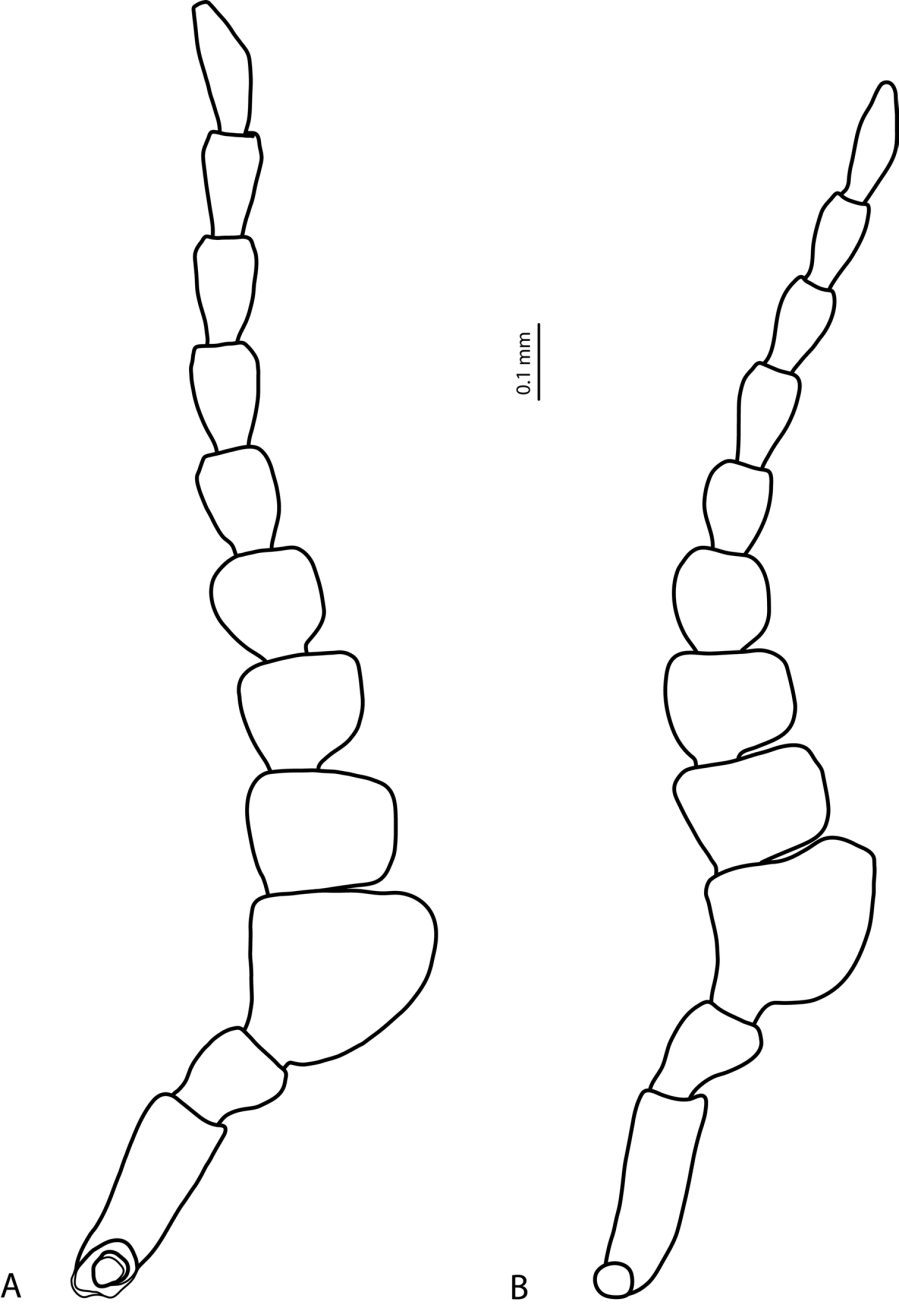
Male antennae **A***Exocelina athesphatos* sp. nov., paratype **B***E. tsinga* sp. nov., paratype.

**Figure 9.**
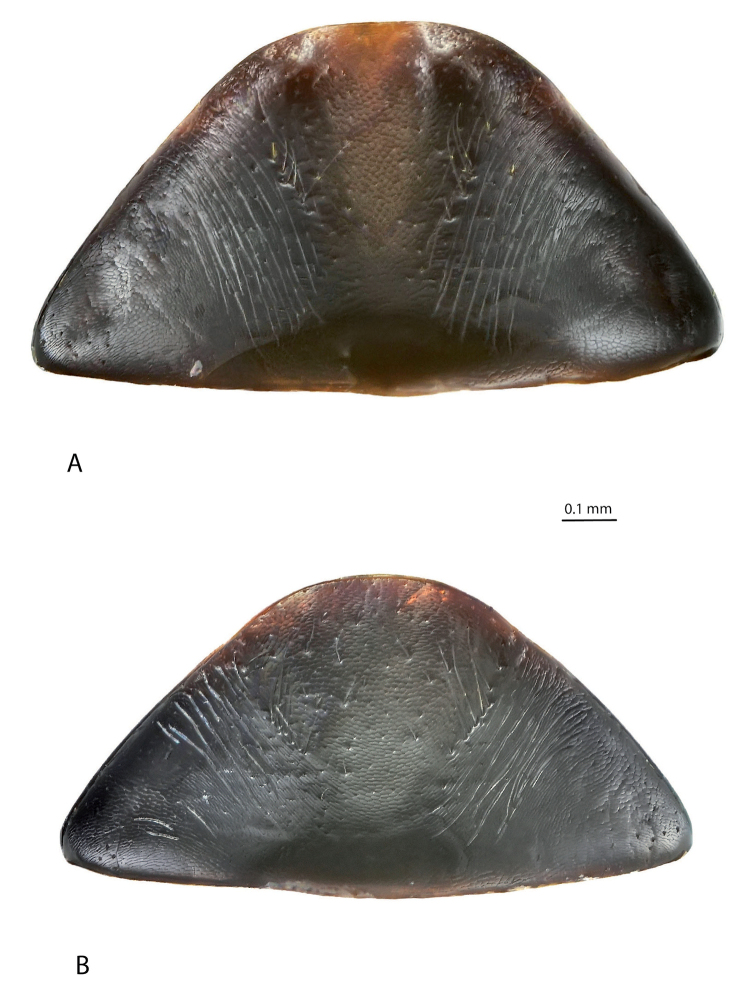
Abdominal ventrite 6 **A***Exocelina athesphatos* sp. nov., paratype **B***E. tsinga* sp. nov., holotype.

**Figure 10.**
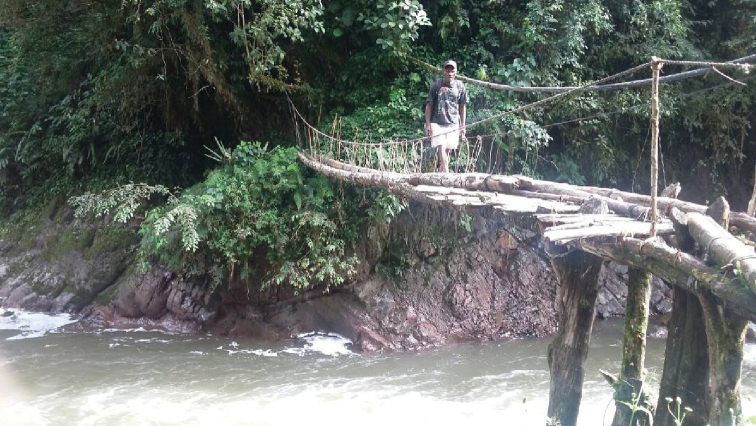
Tsingogong River.

**Figure 11.**
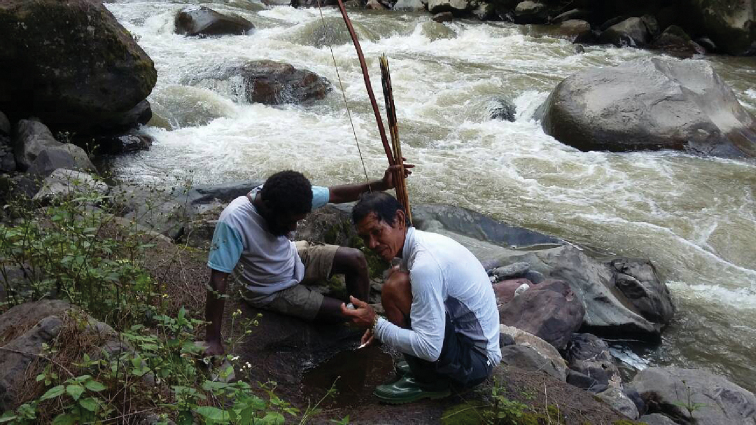
Small water holes on bedrock besides Tsingogong River.

Figures 5 , 6 , 7 , 8B , 9B , 10 , 11

#### Type locality.

Indonesia: Papua Province, Mimika Regency, Tsinga Village, Tsingogong River, 04°11.320'S, 137°16.364'E, 1,306 m a.s.l.

#### Type material.

***Holotype***: male “Indonesia: Kabupaten [Regency] Mimika, Desa [Village] Tsinga, Sungai [River] Tsingogong”, “1306 m, 25–30.v.2017, 04°11.320'S, 137°16.364'E, B. Sumoked” (MZB). ***Paratypes***: 33 males, 38 females with the same label as the holotype (MZB, KSP). 6 males, 7 females “Indonesia: Kabupaten Mimika, Desa Tsinga, 1381 m, 25–30.v.2017”, “04°11.379'S, 137°13.456'E, B. Sumoked” (MZB, KSP).

#### Description.

***Body size and form***: Beetle medium-sized: TL-H 4.05–4.8 mm, TL 4.5–5.3 mm, MW 2.15–2.5 mm (holotype: TL-H 4.5 mm, TL 5 mm, MW 2.4 mm), with oblong-oval to elongate habitus (Fig. 5).

***Colouration***: As in *E. athesphatos* sp. nov.

***Surface sculpture***: As in *E. athesphatos* sp. nov.

***Structures***: Pronotum with distinct, relatively narrow anteriorly lateral bead. Base of prosternum and neck of prosternal process with distinct ridge, slightly rounded anteriorly. Blade of prosternal process lanceolate, relatively broad, slightly convex, with distinct lateral bead and few setae. Abdominal ventrite 6 broadly rounded or slightly truncate.

***Male***: Antenna strongly modified (Figs 5, 8B): antennomere 2 strongly reduced, antennomeres 3 and 4 strongly enlarged (antennomere 3 the largest), antennomeres 5 and 6 distinctly enlarged, antennomeres 7–9 stout. Pro- and mesotarsomeres 1–3 dilated. Protarsomere 4 slightly dilated, with anterolateral angle shortly expanded and with large, thick, slightly curved anterolateral hook-like seta. Protarsomere 5 ventrally with anterior row of 14 and posterior row of 6 short, thick, pointed setae (Fig. 7B). Median lobe relatively long and slender, with slightly discontinuous outline in subapical part (mainly visible in ventral view); in lateral view, apex thin, more or less pointed and curved downwards; in ventral view, apex broad, almost truncate (Fig. 6). Paramere with very deep dorsal notch, separating subdistal part; subdistal part is large, broad, curved downwards and pressed closely to paramere, with fringe of seven very broad, flattened setae; proximal setae numerous, dense, thin, much more inconspicuous than subdistal (Fig. 7A). Abdominal ventrite 6 slightly depressed medially, with 10–12 lateral striae on each side (Fig. 9B).

***Female***: Antennae and pro- and mesotarsi not modified. Abdominal ventrite 6 without depression and lateral striae.

#### Affinities.

About the placement within the *E. ekari* group, we consider the species in the same way as *E. athesphatos* sp. nov. Both species are very similar in external morphology (colouration, body form and sculpture, shape of male antennae) and, therefore, difficult to distinguish without detailed study (Figs 1, 5). However, males antennomeres of *E. tsinga* sp. nov. are slightly smaller than in *E. athesphatos* sp. nov. and have slightly different form (Fig. 8). The species can be easily separated by the shape of abdominal ventrite 6 (Fig. 9), median lobe and paramere.

#### Distribution.

Indonesia: Papua Province, Mimika Regency. The species is known only from the type locality.

#### Habitat.

The specimens were collected from small puddles on bedrock (Fig. 11), besides fast flowing mountains streams (such as Tsingogong River in Fig. 10).

#### Etymology.

The species is named after Tsinga Village. The name is a noun in the nominative singular standing in apposition.

## Supplementary Material

XML Treatment for Exocelina athesphatos

XML Treatment for Exocelina tsinga
